# Community-informed research on malaria in pregnancy in Monrovia, Liberia: a grounded theory study

**DOI:** 10.1186/s12936-018-2529-5

**Published:** 2018-10-23

**Authors:** Christine K. Tarr-Attia, Quique Bassat, Bondey Breeze-Barry, Dawoh Peter Lansana, Ana Meyer García-Sípido, Adelaida Sarukhan, Maria Maixenchs, Alfredo Mayor, Guillermo Martínez-Pérez

**Affiliations:** 1 Saint Joseph’s Catholic Hospital, Tubman Boulevard, Oldest Congo Town, PO Box 10-512, 1100 Monrovia, Liberia; 2 ISGlobal, Hospital Clínic - Universitat de Barcelona, Barcelona, Spain; 3 Centro de Investigação em Saúde de Manhiça (CISM), Maputo, Mozambique; 4 ICREA, Barcelona, Spain; 5 Hospital San Joan de Déu, Barcelona, Spain; 6 NGO Juan Ciudad Foundation, Madrid, Spain

**Keywords:** Malaria, Liberia, Qualitative research, Pregnancy, Prevention, Access to healthcare, Feminist grounded theory

## Abstract

**Background:**

Liberia is a West African country that needs substantial investment to strengthen its National Malaria Control Programme (NMCP), which was disrupted during the 2014–2016 Ebola epidemic. As elsewhere, Liberian pregnant women are especially vulnerable to malaria. Understanding prevention and treatment-seeking behaviours among the population is crucial to strategize context-specific and women-centred actions, including locally-led malaria research, to improve women’s demand, access and use of NMCP strategies against malaria in pregnancy.

**Methods:**

In 2016, after the Ebola crisis, a qualitative inquiry was conducted in Monrovia to explore populations’ insights on the aetiology, prevention and therapeutics of malaria, as well as the community and health workers’ perceptions on the utility of malaria research for pregnant women. In-depth interviews and focus group discussions were conducted among pregnant women, traditional community representatives and hospital staff (*n *= 38), using a feminist interpretation of grounded theory.

**Results:**

The narratives indicate that some Liberians believed in elements other than mosquito bites as causes of malaria; many had a low malaria risk perception and disliked current effective prevention methods, such as insecticide-treated nets; and some would resort to traditional medicine and spiritual care to cure malaria. Access to clinic-based malaria care for pregnant women was reportedly hindered by lack of financial means, by unofficial user fees requested by healthcare workers, and by male partners’ preference for traditional medicine. The participants suggested that malaria research in Liberia could help to design evidence-based education to change current malaria prevention, diagnostic and treatment-seeking attitudes, and to develop more acceptable prevention technologies.

**Conclusion:**

Poverty, insufficient education on malaria, corruption, and poor trust in healthcare establishment are structural factors that may play a greater role than local traditional beliefs in deterring Liberians from seeking, accessing and using government-endorsed malaria control strategies. To increase access to and uptake of preventive and biomedical care by pregnant women, future malaria research must be informed by people’s expressed needs and constructed meanings and values on health, ill health and healthcare.

## Background

Malaria is a parasitic disease transmitted to humans through mosquito bites. Pregnant women are at most risk of malaria disease [1]. In spite of a reduction in malaria-attributable morbidity and mortality among this vulnerable group [1], current global malaria control efforts are threatened by the spread of multi-drug resistant parasites [2, 3], pyrethroid-resistant mosquitoes [3, 4], interruption of free healthcare in some endemic settings [5], and cultural beliefs and gendered norms that deter women from accessing biomedical care against malaria disease [6–9]. Universal access to malaria prevention and care for African pregnant women is yet to be achieved [1, 10].

Liberia, in West Africa, is a country that needs investment to strengthen its national malaria control program (NMCP). According to the last 2016 Malaria Indicator Survey (MIS), prevalence of *Plasmodium falciparum* infection among 6–59 months-old children in Liberia was as high as 45% [11]. The 2016 MIS did not report data on malaria prevalence among pregnant women. The recent 2014–2016 Ebola epidemic in Liberia decimated its health workforce and compromised healthcare provision for patients suffering from malaria and other infectious diseases [12, 13]. It is estimated that the disruption of routine healthcare provision might have led to increases of up to 62% in the rate of malaria-attributable mortality [14].

The Liberian Ministry of Health and Social Welfare (LMoHSW) recommends intermittent preventive treatment during pregnancy (IPTp); provision of insecticide-treated nets (ITN); and indoor residual spraying (IRS) to prevent malaria [15, 16]. However, according to the 2013 Demographic and Health Survey (DHS), only 37% of pregnant women slept under an ITN the night before the survey [17] and approximately 48% received two or more doses of sulfadoxine–pyrimethamine as IPTp in the preceding 2 years [17]. These data show that there is room for improvement in the scaling-up of strategies to protect pregnant women from malaria.

The World Health Organization (WHO) Global Technical Strategy for malaria highlighted the need to understand people’s prevention and treatment-seeking behaviours in high transmission settings [18]. On one hand, socio-economic and culturally mediated constructs on health and disease influence how pregnant women understand and use malaria care for themselves and their progeny [19–21]. In addition, structural contributors such as poverty, illiteracy, food insecurity and political instability also affect malaria care-seeking behaviours and contribute to malaria burden [21–23].

There is need to strengthen the implementation of NMCP strategies to protect pregnant women from malaria in a post-Ebola scenario. Promotion of opportunities for local health and social researchers, public health experts and frontline healthcare workers to jointly plan, conduct and evaluate research on what factors may be driving some pregnant women not to seek for, access or appropriately use and adhere to LMoHSW and WHO-endorsed prevention and treatment interventions, is important to better design strategies aimed at strengthening NMCP programmes. Gathering sociocultural information on populations’ experiences with malaria in a post-Ebola scenario is key to inform, among others, the design of research aimed at developing innovative malaria prevention tools, the planning of communication activities to reinforce populations’ malaria care-seeking behaviours, and the monitoring of how current NMCP-endorsed interventions are accepted by the population and effectively integrated by healthcare professionals in their routine practice. However, in spite of the variety of socio-epidemiological studies on Africans’ understanding of malaria [20, 21], there is a scarcity of such studies in Liberia.

This article reports on a study conducted in Monrovia, Liberia’s capital, with the overall purpose to gather information on the barriers and opportunities for pregnant women to participate in malaria research. A specific objective of this study was to gather culture-grounded information to assess insights on the aetiology, prevention and therapeutics of malaria among Liberian communities, as well as communities’ perceptions on the utility of malaria research for pregnant women. It is expected that the information obtained will guide the design of future malaria research aimed at proposing culturally-congruent ways to ensure that all pregnant women benefit from any of the NMCP-endorsed strategies to prevent and treat malaria.

## Methods

### Design and site

This was a qualitative study in design that was conducted between November 2016 and May 2017 alongside a larger cross-sectional assessment of the prevalence of malaria among pregnant women attending first antenatal care at the Saint Joseph’s Catholic Hospital (SJCH). The SJCH is a not-for-profit hospital established by the Hospitallier Order of the Brothers of Saint John of God in Congo Town neighbourhood, Monrovia, in 1963.

In mid-2016, the SJCH, in cooperation with Barcelona Institute for Global Health (ISGlobal), offered training in basic fundaments of medical research ethics to a group of traditional community representatives from Congo Town. All trainees were invited to constitute a Community Advisory Board (C.A.B). Prior to implementing this study, advice from the C.A.B was sought with regards to the appropriateness of the informed consent and data collection procedures.

### Sampling and recruitment

The study population comprised three key-informant groups (KIG):

KIG1: Pregnant women who had previously participated in the malaria prevalence study (hereafter the *women*). A list produced from the prevalence study dataset, was used to randomize and invite *women* as potential participants for KIG1. Study participants younger than 18 years were excluded from the randomized list.

KIG2: Traditional community representatives (the *leaders*). With assistance from the SJCH Social Work Department, potential participants for KIG2 were identified among the traditional authorities, key community influencers and other community representatives within Congo Town neighbourhood.

KIG3: SJCH medical, laboratory and management staff (the *staff*). During the study period, the SJCH had over 170 staff. The social scientist (GMP) approached potential participants for KIG3. SJCH staff engaged in the planning and implementation stages of the prevalence study were not involved as KIG3.

Patton [24] and Charmaz [26] recommendations to sample information-rich informants from which an in-depth understanding of the study phenomena could be obtained were followed. Approached individuals were not sampled by proximity or availability. Rather, by theoretical sampling: a grounded theory instrument which involves inviting informants who could potentially co-generate data that could further improve understanding of theoretical categories, information and interpretations co-generated during previous encounters with enrolled study participants [26].

Potential participants were either phoned or contacted face-to-face. The study purpose was explained to all approached individuals, and, if interested, an invitation to meet the research team was offered. At the scheduled date, written informed consent to take part in an in-depth interview (IDI) or in a focus group discussion (FGD) was obtained. All individuals were informed about the measures in place to protect their confidentiality. Participants were reminded of their right not to answer any question that they did not wish to, and their right to withdraw from the study at any time. Participants to the FGDs were reminded not to repeat any of the other participants’ comments outside the group.

All participants received a grocery voucher of 10,00 USD. Recruitment was discontinued when theoretical saturation—whereby all themes had been thoroughly explored in detail and no new themes kept emerging in subsequent data collection—was achieved [25].

### Data management and analysis

IDIs and FGDs were conducted by a European white male social scientist (GMP), aided by a Liberian black female co-researcher nurse (CKTA) who worked at the SJCH and, hence, decided not to participate in the KI3 group interviews. During data collection, no one else but the interviewers were present. The majority of interviews took place at the SJCH premises.

A thematic interview guide was used with all KIGs. However, the manner in which questions were formulated to *women*, *leaders* and *staff* varied. More sensitive questions (e.g. personal beliefs in witchcraft) were reserved for the IDIs.

Interviews were held in English and were tape-recorded. Consent documents were stored in a locked cabinet at the SJCH and received a Unique Identification Number to enable their linkage to the participants’ recordings. All recordings were transcribed verbatim into a MS Word document. Transcriptions were de-identified and cross-checked against the recordings. All transcripts were imported into password-protected Dedoose software (^®^SocioCultural Research Consultants, Manhattan Beach, CA). After data analysis, all recordings were deleted.

Transcripts were coded in an iterative manner with data generation to ensure that all core concepts were addressed during the conduct of the IDIs and FGDs. Data were line-by-line coded at first, using open coding and gerunds [26, 27], to develop a coding framework that was used to code all the transcripts.

A feminist interpretation of grounded theory guided this study [28–30]. This approach implies that: (a) women participants are addressed as ‘co-generators’ of data and theory in cooperation with the social scientist, who (b) must practice reflexivity (i.e. memo writing to reflect on the impact of the research process and of her/his own interaction with the participants [27]), (c) must be sensitive towards issues of oppression and marginality that might be affecting the participants during the research process, (d) must ensure the findings of the research are useful to improve women’s health, and (e) must promote social change [28–30].

To ensure trustworthiness, triangulation was done at several levels. Firstly, three different groups of key informants were invited to partake in data generation. Secondly, interim analysis of deviant cases was done and narratives from the IDIs were triangulated with narratives from the FGDs. Thirdly, upon completion of data coding, self-reported views were compared with findings from similar studies in the region. Once the study concluded, the SJCH C.A.B was invited to peer-review the research team’s interpretation of the participants’ narratives.

This article has been prepared as per reporting standards set forth in the COREQ checklist [31]. With the aim of respecting the participants’ perspectives, concepts and notions are reported using their own words in *‘italics’.* Excerpts have been edited because most participants used *‘colloquia’* (i.e. Liberian English [32]) during data collection. As per the methodological package chosen to guide conduct of this research, the interpretations of the phenomena under study that are reported in the “Methods” section of this article are consistent with the participants’ voices [26–30].

### Ethics approval

The study received approval from the University of Liberia-Pacific Institute of Research & Evaluation Institution Review Board (Monrovia, Liberia) and from the Hospital Clinic Research Ethics Committee (Barcelona, Spain).

## Results

Seventeen *women*, 11 *leaders*, and 10 *staff* consented to participate in 26 IDIs and in three FGDs (Table 1). No approached individual refused to participate. Nine of the participants (22.5%) were men. The median age was 35 years. Twenty-nine participants were born in Montserrado County (Monrovia). Sixteen (40%) and 11 (27.5%) participants had completed secondary and university education, respectively.

**Table 1:** Basic socio-demographics of key informants (KI)

	Recording#		Sex	Current occupation	Age group	County of birth	Education
	IDI	FGD					
KI1: pregnant *women* participants in malaria prevalence study	ni	FGD3	F	Student	16–20	Montserrado	Secondary
	ni		F	Student	31–40	Montserrado	None
	ni		F	Student	21–30	Montserrado	Secondary
	ni		F	Business	31–40	Margibi	Vocational
	ni		F	Student	31–40	Sinou	College
	IDI13	ni	F	Student	21–30	Montserrado	Secondary
	IDI14	ni	F	Student	21–30	Rivercess	Secondary
	IDI17	ni	F	Business	21–30	Montserrado	None
	IDI18	ni	F	Student	21–30	Montserrado	Secondary
	IDI19	ni	F	Business	21–30	Bong	Secondary
	IDI20	ni	F	Business	21–30	Montserrado	Secondary
	IDI21	ni	F	Student	21–30	Montserrado	Secondary
	IDI22	ni	F	Student	21–30	Nimba	Secondary
	IDI23	ni	F	Physician assist.	21–30	Montserrado	College
	IDI24	ni	F	Student	21–30	Grand Cape	Secondary
	IDI25	ni	F	Business	21–30	Grand Bassa	Primary
	IDI26	ni	F	Student	21–30	Montserrado	Secondary

	Recording#		Sex	Traditional role	Age group	County of birth	Education
	IDI	FGD					
KI2: traditional community representatives	IDI1	FGD2	M	Youth leader	31–40	Montserrado	Secondary
	IDI10		F	Chairlady	41–50	Montserrado	None
	ni		M	Council of elders	41–50	Montserrado	University
	ni		M	Council of elders	51–60	Montserrado	Secondary
	ni		F	Chairlady	31–40	Montserrado	University
	IDI2	ni	F	Chairlady	51–60	Montserrado	Secondary
	IDI3	ni	M	Chief	41–50	Montserrado	University
	IDI8	ni	F	Chairlady	41–50	Montserrado	Secondary
	IDI9	ni	F	Trad. midwife	61–65	Montserrado	Vocational
	IDI11	ni	M	Herbalist	51–60	Montserrado	Secondary
	IDI12	ni	M	Council of elders	51–60	Montserrado	College

	Recording#		Sex	Department	Age group	County of birth	Education
	IDI	FGD					
KI3: SJCH Medical, Administrative and Laboratory *staff*	IDI5	FGD1	F	Medical	31–40	Montserrado	University
	ni		M	Administrative	51–60	Montserrado	University
	ni		F	Medical	31–40	Montserrado	University
	ni		F	Medical	21–30	Montserrado	University
	ni		F	Medical	31–40	Montserrado	University
	IDI4	–	M	Laboratory	31–40	Montserrado	University
	IDI6	–	M	Medical	41–50	Grand Bassa	University
	IDI7	–	F	Medical	31–40	Montserrado	University
	IDI15	–	F	Medical	31–40	Nimba	College
	IDI16	–	F	Medical	51–60	Montserrado	College

*ni* not interviewed: participants who only partook either in an IDI or in a FGD

### Identifying the causes of malaria

All participants expressed that every Liberian should know that mosquitoes transmit malaria. Nevertheless, in addition to mosquito bites, the participants explained that some people might believe that malaria may be caused by other vectors, environmental conditions and supernatural agents. No participant mentioned any specific belief on causes of malaria during pregnancy. Only two participants, one *staff* and one *leader,* mentioned that some people may believe that *‘beer’* consumption and ‘*eating too much oranges and plums’* are possible causes of malaria:

*They don’t understand that mosquitoes come to damp places. Most of the time during the rainy season. Or during the time the plum will fall. So when they go to pick up the plums, sometimes they get bitten by mosquitoes!* **(IDI5)**

No participant thought that malaria could be caused by *‘witchcraft’* or by *‘evil water spirits’* such as the *‘Jinne’*. However, *leaders* and *staff* remarked that some people, both in Monrovia and in rural areas, might believe that these entities could be causes of malaria. One *staff* explained how *‘witchcraft’* causes symptoms similar to malaria:

*Malaria attacks the blood vessels. According to their belief, if a witch is in their community, that witch is attacking the blood vessel also. So the witch is sucking their blood. And malaria is also sucking the blood.* **(FGD1)**

One *staff* criticized that the malaria prevention campaigns she had seen failed to explain the consequences of delaying treatment and, as a result, developing *‘cerebral malaria’*. According to this *staff*, lack of information on how malaria disease progresses reinforced the belief that *‘cerebral malaria’* was provoked by *‘witch’*. One leader explained that people delayed seeking for clinic-based care in order to avoid being *‘stigmatized’* by other community members who might think they are *‘affected by witch’*.

Vectors other than mosquitoes were mentioned by *leaders* and *women* as causes of malaria. Cockroaches and rats were considered malaria carriers because, similar to mosquitoes, they *‘play in the garbage and in the water that you drink’*. In addition to these animals, all participants mentioned that they believed in environmental causal factors for malaria, particularly the fact of living in Monrovia, a city surrounded by a *‘swamp’*, where there are many *‘open drainages’*, and where people do not bother to remove the *‘dirt’*.

Because there might be a variety of unfounded beliefs on the causes of malaria, research on the mosquito populations in Liberia was advised by some participants as a helpful means to obtain information useful to sensitize the population that the *Anopheles* mosquito is the only malaria parasite carrier. One male *leader* suggested to investigate why, after the Ebola crisis, the mosquitoes seemed to have *‘changed their form’* referring to the itchiness of the bite. One *staff* and another *leader* suggested studying the mosquito population in order to inform the communities why *‘some mosquitoes [that bite] do not transmit malaria’*.

### Preventing malaria in Liberia

The participants reflected on how uptake of preventive measures was influenced by what the communities perceived as causes of malaria. All participants complained that ITN were not distributed *‘freely’* as they were in the past and that, as one *leader* put it, the population *‘hate them!’* Various reasons were provided for ITN poor acceptability. Sometimes the ITN were used to cover the ‘*dead bodies’* and the relatives refused to use them afterwards. Others expressed how people may complain that the *‘scent can give them flu’,* and that the *‘chemicals burn their skins’*. That was the reason why some people may *‘wash the chemical out’* and use the nets, once cut into smaller pieces, for other purposes (e.g. as sponges for body hygiene). The most common complaint expressed by the study participants was that the nets *‘give heat’*. One *staff*, who complained that at the hospital many patients refused to sleep under an ITN, summarized the reasons why people dislike ITN:

*Mosquito net has a lot of heat. People’s houses are so close! They don’t have windows to breath. So if you don’t have windows, a ventilated place, if you put a mosquito net, people will suffocate. And another reason is that the net itches. It can burn! They say ‘put it in the sun for ‘x’ amount of hours or days before you can put it’. We have no patience for that! So many reasons why we don’t use mosquito nets! …. every morning you untie it, every evening you tie it up. A lot of time. And energy! And we come home tired. And every time you want to come out to urinate, you have to lift it up… too many complications!* **(IDI5)**

The participants described how partners’ attitudes might impede some women to use ITN. One *woman* expressed that she would *‘sleep on a mosquito net, but in the dry season he takes out the net’*. All *women* explained how they had already taken IPTp which, as one *staff* complained, was currently the only free prevention that pregnant women could receive at their antenatal care visits.

No participant used or knew of any traditional *‘country medicine’* to avoid malaria. Only two *staff* thought that some people may *‘chew cola nut’* and *‘burn leaves from the bush’* in their rooms to chase the mosquito away. One male *leader*, an *‘herbalist*’, confirmed that his *‘ancestors’* did not teach him any prevention.

Other vector control means were described. Indoors, some people used electric fans. However, the majority of communities lacked constant power supply and, hence, used windows screens, insecticide spray and anti-mosquito coils instead. Outdoors, the participants’ communities tried to clean their environment and get rid of dirty stagnant water. A common complaint from the participants was that environmental control depended entirely on the communities, which were unassisted by the authorities. No participant knew of IRS conducted by any organization. Only one *leader* described how there were community members who ‘*pass by, we pay them fifteen USD, and they spray your house’*.

In spite of people being knowledgeable on what to do to prevent malaria, some participants perceived that malaria was still highly prevalent in Liberia because *‘people are just care*-*free’*. In this regard, a common perception was that dissemination of research findings could motivate the communities to use the most effective prevention means. In agreement with the *leaders* in one FGD, one *staff* explained how information from research could help people to understand that some of their preventive behaviours (i.e. burning *‘rubber tyres’* to drive mosquitoes away) were actually harmful to their health.

Future research, as all *staff* suggested, should prioritize development of new prevention strategies. One *staff* gave some ideas of prevention means that, if developed in the context of research in Liberia, he thought could be widely accepted:

*For the pregnant women […] you get prophylactic IPTp. So, if they can find another IPTp for children or for adults who are not pregnant, that would also help. And the kind of materials that you use in the insecticide net, if it were possible to put them to some lotion that you can rub.* **(IDI5)**

### Suspecting and diagnosing malaria

Fever, chills, vomiting, and weakness were identified as symptoms that helped people suspect they could be developing malaria. Other less common signs mentioned were loss of appetite, constipation, diarrhoea, itchiness, tremors, coughing, and *‘a bitter taste in your mouth’*. No participant mentioned any symptom or sign particular to malaria in pregnant women.

The participants distinguished *‘simple’* or *‘common malaria’* from what they alternatively termed *‘chronic’*, *‘severe’*, *‘hard’*, *‘brain’* or *‘3*+*/4*+* malaria’*. No participant mentioned that pregnant women could be affected by *‘chronic’* malaria to the same degree as children. According to their narratives, when parents delayed initiating treatment for their children, *‘their brain goes wrong’* and untreated children could be seen *‘convulsing’*, *‘going off’*, and *‘collapsing’.* One *leader* explained that severe malaria could make children *‘go blind, deaf and dumb’*. As one *staff* explained, some parents may believe the children are *‘bewitched’* rather than affected by malaria.

Passive case detection was described as the most usual approach to diagnose malaria. Reportedly, pregnant women would usually go to a clinic or, alternatively, to a *‘drugstore’* to receive a malaria test. There was consensus that no woman would be afraid to discover that her *‘malaria status is positive’*. The *women* explained that some pregnant women might ask for their partners’ permission to test for *‘stigmatizing* conditions’ such as HIV/AIDS, Ebola, and tuberculosis, but not for malaria. Hence, irrespective of the testing site, the major reported barrier for pregnant women to obtain a malaria diagnostic was the test’s cost.

As some women could fear the *‘pricking’* of the lancet, the *leaders* in one FGD suggested that research could be useful to develop painless *‘saliva’*-based tests. For future research, the same *leaders* suggested to always use point-of-care tests because many women might suspect that their blood specimens—if taken by syringe to be tested somewhere else—could be *‘sold’* by the researchers; used to *‘develop new viruses [like Ebola]‘*; used to do *‘witchcraft’*; or used to test them for HIV/AIDS against their will.

Throughout the IDIs and FGDs, complaints about the general population not paying the necessary attention to detect signs of malaria kept emerging. In this regard, one female *staff,* who explained that some parents arrive in the hospital with their children ill with *‘4*+* malaria’* because they had *‘neglected their responsibility’* to demand a malaria RDT when the symptoms started, suggested that parents’ healthcare and diagnostic-seeking attitudes should be explored in the frame of research. Findings from malaria research in Liberia could be used, according to the participants’ suggestions, to design health education campaigns for women, to emphasize the need to seek hospital care when unsure of what condition might be affecting them or their children.

### Accessing malaria treatment at clinics

The majority of participants considered pregnant women as an especially vulnerable population because of the multiple barriers to access healthcare that they face. Lack of government-run healthcare facilities in many urban and rural settings; *‘inefficacious’* drugs to treat malaria in pregnancy; men who *‘force’* their partners to go to the *‘herbalists’*; and lack of competence on malaria care of *‘traditional midwives’* were mentioned as factors contributing to pregnant women’s vulnerability to malaria in Liberia.

The major perceived problem for pregnant women to access malaria treatment was its cost. The *staff* expressed that, in order to avoid pre-term birth and low birth weight, free access of pregnant women to malaria care had to be ensured. Everybody but one *woman*—an LMoHSW physician assistant—agreed that, even if government-run facilities were expected to provide *‘free care’*, many women ended up paying *‘little somethings’* to the healthcare personnel or being sent to the private *‘drugstores’* because the clinics had run out of anti-malarials.

Some participants described how some women, discouraged by these situations, could prefer to seek malaria care at the *‘herbalists’* or at the *‘black dealers’* (street vendors). The *leaders* and *women* in FGD2 and FGD3 were clear that many women’s first choice to treat malaria would be clinic-based biomedical care and that many would resort to *‘country medicine’* only when adverse circumstances (i.e. long-distances to nearest clinic; lack of financial means to pay unofficial fees) forced them to.

The LMoHSW physician assistant explained that, as per national policies, Quinine was given to pregnant women in their first trimester only, and that artesunate–amodiaquine (AS–AQ) was prescribed in their second and third trimesters as the first treatment choice for uncomplicated malaria in adults. A few participants mentioned that they themselves disliked Quinine because it causes weakness, dizziness, and, especially, a *‘ring bell in the ear’*. According to some *women* and *leaders* who recalled their own experiences as patients, anti-malarials other than AS–AQ were easily accessible over-the-counter for the population. *Lokmal*^®^ (artemether–lumefantrine) was referred to as the preferable treatment for those people who disliked AS–AQ. Amodiaquine, popularly referred to as *‘amgonnakillyou’* (“I am gonna kill you”), was especially feared due to its perceived side effects:

*My sister died of Amodiaquine. When she went to the hospital, they gave it to her. She threw up. And then she took the same prescription again. Then she became very weak. By the time we took her to the hospital, the staff said she overdosed herself.* **(FGD2)**

There was consensus that *‘people don’t take malaria seriously’*. This perception was sustained in that, quite often, when women felt that they or their children had fever, they would buy and consume anti-malarials or *‘headache medicine’* in the absence of a confirmatory malaria test. These medicines can be directly bought from the boys *‘selling them in their buckets’* in Monrovia. One *leader* narrated her own experience administering antipyretics to her daughter:

*I was giving [my daughter] paracetamol. She went to school. In the night her skin got hot! So I said: ‘Maybe the blood is low, so let me buy some blood tonic’. Later on, I saw my in*-*law working here at SJCH. I told him: ‘You got to go to the house and give [my daughter a] drip’. He said: ‘No, you have to bring her to the hospital’. I said: ‘I don’t have money’. So he said: ‘Bring her’. Because some time ago, they had free treatment for children. So I brought her, they did the test, she had 2*+ *malaria.* **(IDI10)**

Precisely because some pregnant women may have difficulties to pay for malaria care, some participants, especially the *leaders*, thought that a potentially effective approach to encourage them to participate in research would be to tell them, in the frame of future community mobilization activities, that *‘free tests and treatment’* are provided to research participants.

### Using traditional medicine and spiritual care to treat malaria

According to the participants, *‘country medicine’* was highly valued in rural areas. One *staff* explained that many patients would arrive in the hospital only when ‘*country medicine’* had not proven effective. The *staff* reported that patients would not disclose that they had sought assistance from the ‘*herbalist’* but that, however, their use of *‘country medicine’* would be revealed as soon as ‘*a nasogastric tube is put and the herbs come out of the stomach’*.

One *leader*, a *‘herbalist’*, explained how he acquired knowledge on which *‘herbs’* from the *‘bush’* he could use from his *‘forefathers’*. According to his narratives, the herbs were mixed *‘depending on the type of malaria. Simple malaria: you don’t mix them unless the malaria is chronic in you’.* Some participants agreed that one of the reasons why the *‘herbalists*’ were appreciated by the Liberian population was because the *‘herbalists’* tried their ‘*mix of herbs’* on themselves before administering them to their clients. Hence, a suggestion made by one *leader* was that researchers should also try the experimental drugs and vaccines in the presence of the pregnant women approached as study subjects.

Some participants reflected that the *‘herbalists’* were also appreciated because they did not charge their clients unless their ‘mix of *herbs’* had worked. However, there was no consensus among the participants on whether the ‘*herbalists’* fees were higher or lower than the *‘little somethings’* that people were, allegedly, asked to pay at government-run facilities. In addition to the *‘herbalists’*, a few participants complained that some people may ask for assistance to certain *‘prayers’* (i.e. unspecified evangelical religious leaders) that were offering spiritual care to release *‘evil spirits’*. Some *leaders* expressed concern about the consequences for children who develop cerebral malaria and whose parents decide to take them to the *‘prayers’*:

*We all know that malaria causes a child to jerk, right? You see them going off and scratching. They say: ‘An evil spirit came’. And then they take him to the prayer. They will not give you pills. They only pray. And that person [the child] will be there until he dies.* **(FGD2)**

*Staff* and *leaders* described how people without means to pay *herbalists’* or *‘preachers’* self-treated with locally available herbs, such as ‘*sugarcane leaves’* or ‘*moringa’*. *Leaders* in FGD2 described how the Kru tribe ‘*beat pepper, rub it on the back, put it in the anus.’* The expected function of these home remedies was to help the body expel the malignant force through the skin pores:

*I go to the bush, pick my herbs, put them in the fire, and boil them. That’s how I’ll be treating myself. Because when the malaria is chronic in you, the whole body becomes cold. I am not sweating because my pores aren’t open. I will not sweat, except if I drink the herbs. So I boil them and heat myself. I sit in a chair, and cover myself with a blanket. As I’ll sweat now, I’ll be free from malaria.* **(IDI11)**

The *staff* complained that some parents—whether by own initiative or following an *‘herbalist’s’* advice—used *‘herbs’* and *‘ointments’* to *‘bathe’* the children to subdue the fever. However, the *staff* expressed that nowadays, thanks to *‘improved education’*, it was not that frequent that parents arrived in the hospital with their children with *‘4*+ *malaria’* after they had been administering them local remedies.

*When there is fever, they boil some leaves and bathe the children with the leaves. Or boil some roots and you drink the root. Each culture has its way of treating it. For example, some people will use butter pear leaves mixed with tea leaves. And some […] rub the herbs on the body.* **(IDI6)**

To better sensitize women on ‘*what can happen’* to children and themselves when infected with malaria, some *staff* proposed further studies on ‘*pathophysiology of malaria’* to be conducted in Liberia. To help pregnant women prioritize clinic-based malaria care, it was also proposed that the efficacy of traditional remedies be investigated. The *leaders* manifested that dissemination of findings of malaria research demonstrating the ineffective effects of *‘country medicine’* consumption and the adverse effect of delaying visits to the clinic could be more effective in raising awareness among pregnant women to first resort to clinic-based malaria care.

## Discussion

Liberians’ perspectives on the aetiology of malaria and on the means to prevent and treat this disease could be mediated by traditional beliefs and influenced by their most immediate socio-economic environment. Poverty, local beliefs regarding the causes of ill health, insufficient exposure to health education, corruption in the health system, and populations’ acquiescence with non-biomedical malaria treatment providers such as *‘herbalists’*, *‘prayers’* and street drug sellers are, as per the study participants’ narratives, among the elements that most influence pregnant women’s decisions on preventing and caring for malaria.

The anthropologist Linda Collier Jackson, in a study published in 1985, reported how, when malaria infection caused protracted illness in children and mothers, Liberians felt that there were underlying spiritual factors that led them to consult a traditional doctor to obtain herbal preparations and ritualistic directives [33]. To the authors’ knowledge, no other anthropological study on malaria in Liberia has been published since 1985. Hence, the findings from this study may represent the first grounded theory study conducted in the country shortly after the Ebola epidemic ended. Many of the study findings do not differ significantly from other African settings. For example, indirect causal factors—such as stagnant waters and unhygienic surroundings—were identified as direct causes of infection, as reported for Burkina Faso [34], Gambia [35, 36], Ghana [21], Malawi [21], Nigeria [37] and elsewhere. Witchcraft as a perceived cause of malaria-like febrile diseases was also reported in The Gambia [35], Gabon [38], and Ivory Coast [39] and even in distant regions such as Panama [23]. Perceptions that individuals presenting with malaria-related symptoms (i.e. fever, unconsciousness and convulsing) may suffer from witchcraft or attacks from evil spirits, have been previously documented in Tanzania [40, 41] and in The Gambia [35].

As in other African countries [34, 42, 43], the participants expressed how the nuisances associated to the ITNs condition the population’s willingness to use them. This finding, however, contradicts a previous study in Liberia reporting good acceptability of long-lasting alpha-cypermethrin-treated ITNs [44]. Gendered norms that determine use of ITNs within households and that make some women accept the therapeutic methods imposed by their male partners have also been documented elsewhere [6, 7, 34].

It must be noted that in Liberia, some of the behaviours reported in this study are not exclusively driven by traditional beliefs or gendered constructs. Rather, they are the product of structural factors with no short-term solution. Behaviours that put pregnant women at increased risk of the harmful effects of malaria disease may be the consequence of household economic circumstances and of the multiple impediments to access clinic-based care in a public healthcare system that has suffered from chronic mismanagement. As suggested by some participants, risky behaviours may also be the consequence of the government’s insufficient investment in access to health education for women.

Lack of financial autonomy could also make women accept their partners’ petitions to disregard certain malaria prevention and care behaviours of proven effectiveness, such as ITNs. As in published literature on Liberian women [45], some female participants in this study voiced out their disagreement with the gendered norms that place them in a disadvantaged position compared to their partners. Nevertheless, as firmly expressed by *leaders* and *women* in this study: it may be only because of their ‘circumstances’—namely, lack of financial resources and illiteracy in health issues and in particular in malaria disease—that some women may ‘turn a blind eye’ to prevention and biomedical care.

An added value to this study is the use of a feminist grounded theory approach to contextualize participants’ views with the aim of gathering culture-grounded information to ensure that future malaria research in Liberia responds to the expressed needs of pregnant women. In this regard, the participants, as co-generators of data, demonstrated awareness of the multiple factors that may oppress pregnant women, challenge their access to malaria care and increase their vulnerability to malaria. Based on this awareness, the participants expressed their perceived usefulness of malaria research and suggested research topics (i.e. new women-controlled malaria preventive tools) and considerations for the conduct of malaria research (i.e. use saliva-based RDT) (see Table 2) that could bring about social change for women [30]. These suggestions could help increase pregnant women’s likelihood to receive evidence-based malaria education, use malaria prevention tools, access the safest malaria care, and participate as full right subjects in malaria research.

**Table 2:** Main suggestions from the study participants

Advices on WHAT to research	Advices on HOW to research
Dietary and biting habits of mosquito populations Temporal-spatial trends: changes in mosquito population (e.g. before vs. after Ebola) Develop new prevention tools based on formulations that are accepted by Liberians (i.e. tablets, lotions) Develop innocuous alternatives to blood-based malaria tests (e.g. saliva-based oral tests) Parents’ attitudes towards seeking malaria care for their children when ill Pathophysiology of malaria with a focus on how it affects pregnant women and children and leads to cerebral malaria Effectiveness of traditional home remedies and herbalists’ mixes of local herbs to cure malaria	Use point-of-care rapid diagnostic tests when drawing blood specimens from malaria research participants Test experimental drugs on researchers before administering them to the study populations Use findings of research to sensitize populations to disregard harmful preventive behaviours (i.e. burning tyres) Stop using country medicine to treat malaria Understand the need to follow health authorities’ recommendations to prevent (e.g. ITN) and cure malaria (e.g. biomedical care) Always return to the communities to share findings of research conducted in Liberia

Nevertheless, for social change to materialize, Liberian authorities must ensure mechanisms to empower pregnant women are in place. In this scenario, empowerment implies a transformation of unequal power relations [46], that would require external resources (i.e. a supportive political environment) and internal capacities (i.e. knowledge, autonomy) [47]. Pregnant women need be equipped with knowledge on their women’s reproductive rights and on their rights as patients. They need to actively participate in decision-taking platforms in malaria prevention and control. And they need to be, legally and in practice, protected against abuse in healthcare system environments. Participatory malaria interventions with demonstrated effectiveness to increase women’s self-esteem and self confidence in preventing and controlling malaria in settings such as Thailand [48] could be tested in Liberia. Additionally, local women-led organizations need resources to, firstly, educate women, from a rights perspective, that they are entitled to demand and receive free malaria prevention and care [15, 16], and, secondly, be present in healthcare institutions where they could monitor and denounce, among other issues, the occurrence of informal payments, institutional neglect of patients, and healthcare staff’s refusal to provide them healthcare. Lastly, and in agreement with Gounder and Chaisson [49], there might be more chances for women empowerment, and thus for social change, if all these measures are not constrained to malaria care services but, rather, that are transversally integrated in all women-centred services such as maternal and child health, reproductive health and family planning. However, all these mentioned measures will not suffice to achieve social change unless strategies to improve governance of healthcare systems and to promote change in patriarchy-oriented academic, research and healthcare organizations’ culture are also proposed. In the future, further research will be necessary to ascertain how pregnant women participate in malaria research with full understanding of their human’s, women’s and research subjects’ rights and how this understanding translates into enhanced adherence to malaria prevention and treatment.

Of concern, this study identifies some practices—such as taking the patients with symptoms of severe malaria to the *‘prayers’* or the use of over-the-counter anti-malarial drugs—that are risk factors for infant and pregnant women’s malaria-attributable mortality. Competition for the scarce patients’ resources between *‘herbalists’*, *‘prayers’*, *‘black market’* drug-sellers and healthcare workers working in private *‘drugstores’* could translate into malaria-illiterate women’s confusion about what the most efficacious prevention and treatments are. The existence of unregulated structures operating in parallel to the public health system needs be addressed by Liberian health authorities.

Folk beliefs and remedies and the barriers to access safe quality care revealed in this study should not be ignored in future malaria research involving pregnant women in Liberia. As Anyanwu [37], Cáceres [23] and Ndoye [20] argued, malaria care-seeking behaviours are socially, historically and economically patterned and need be studied from an epidemiological, anthropological and psychological perspective. To strengthen NMCP interventions targeting pregnant women in countries such as Liberia, participatory research approaches could be useful to grasp the way local knowledge is structured. As in feminist grounded theory, participatory action research aims to work with the disempowered and voiceless to identify patriarchy-driven socioeconomic inequalities and to design interventions aiming at improving vulnerable population’s health and wellbeing through a bottom-up approach [50]. Considering that current prevention means may have low acceptance and that access to diagnosis and treatment is hindered by multiple factors, planning for research on new prevention approaches—as some participants suggested—would require consolidating structures for community decision-taking. Participatory approaches could push a research agenda that is women-centred and that could appeal to pregnant women as targeted study participants. Traditional leaders (i.e. chiefs, chairladies) taking prominent roles as research collaborators may have a positive impact on pregnant women’s engagement as participants. However, and in spite of the usefulness of many of the participants’ suggestions for future malaria research, it is of concern that some *leaders* and *staff* proposed to nudge pregnant women into clinical studies by promising them free diagnosis and treatment services in exchange for their participation. Based on the findings of this research, future sensitization and mobilization interventions targeting local communities and their traditional influencers should include content on research ethics, good participatory practices, and fundaments of health research in order to avoid pregnant women experiencing therapeutic misconception and undue influence to enrol in malaria research in Liberia.

## Limitations

Grounded theory approaches to qualitative research aim to understand and interpret how people construct notions on health and well-being and act upon occurrence of ill-health. Hence, rather than a statistically powered size to achieve generalizability of findings [51], this study sought a group of participants that permitted—thanks to their contributions as co-interpreters of their immediate reality—to reach the point of theoretical saturation [25]. Nevertheless, it is possible that the participants’ responses might have been affected by a social desirability bias, and by the influence of the interviewers’ race, gender, nationality and employment status. The majority of interviews were conducted in the SJCH, and, during the informed consent process, the interviewers presented themselves as healthcare professionals working in an international research team. Thus, the possibility that some participants might have overemphasized their personal experiences with informal payments or with non-sympathetic healthcare workers, when seeking malaria care at Liberian clinics, should not be underestimated.

It must be acknowledged that the participants, as inhabitants of Monrovia, had access to employment and education opportunities, to information and communication technologies, and also to communities where health research had been supported. Many inhabitants of rural areas in Liberia lack access to such opportunities. Although none of the participants was engaged in sensitization campaigns on malaria research prior to their participation, most held some knowledge on research—especially because they lived in communities where multiple Ebola-related trials had been conducted in the past 3 years [52, 53]. Hence, the study findings may only represent the insights of Monrovian individuals who hold some knowledge of research. This study does not represent *staff*, *leaders* and *women* who live outside Monrovia and who have never been exposed to health research.

All relevant emerging themes to achieve the study objective were explored. Regrettably, pregnant women’s potential acceptability of novel interventions that had already been tested in other settings (i.e. bug zappers and electronic repellents) and that might be eventually proposed for Liberia was not explored. Contrary to other similar studies in sub-Saharan Africa [21], specific clinical issues around symptomatology of malaria in pregnancy and around pregnant women’s response to anti-malarial therapies unfortunately remained unexplored.

## Conclusion

This study explored how the Monrovian population’s insights on malaria determine their perception of malaria research as useful to improve the health and wellbeing of pregnant women and their communities. Poverty, disempowerment, lack of health education, and a mismanaged healthcare establishment were identified as structural factors that require immediate action as they may play a greater role than traditional beliefs in deterring pregnant women from demanding, accessing and using government-endorsed malaria control strategies. In spite of generalized distrust towards the health establishment, pregnant women might be interested in participating in malaria research, provided this is perceived as useful and that it is planned, in collaboration with the communities, to address their most immediate prevention and care needs.
